# Associations Between Abdominal Obesity Indices and Nonalcoholic Fatty Liver Disease: Chinese Visceral Adiposity Index

**DOI:** 10.3389/fendo.2022.831960

**Published:** 2022-03-10

**Authors:** Xueyu Chen, Fengxue Shi, Juan Xiao, Fengyan Huang, Fang Cheng, Lihua Wang, Yanli Ju, Yong Zhou, Hongying Jia

**Affiliations:** ^1^ Department of Biostatistics, School of Public Health, Cheeloo College of Medicine, Shandong University, Jinan, China; ^2^ Department of Clinical Skills Center, School of Clinical Medicine, Shandong First Medical University, Shandong Academy of Medical Sciences, Jinan, China; ^3^ Center of Evidence-Based Medicine, Institute of Medical Sciences, The Second Hospital, Cheeloo College of Medicine, Shandong University, Jinan, China; ^4^ Clinical Research Institute, Shanghai General Hospital, Shanghai Jiao Tong University School of Medicine, Shanghai, China

**Keywords:** CVAI, NAFLD, abdominal obesity, visceral adiposity, abdominal obesity indices

## Abstract

Non-alcoholic fatty liver disease (NAFLD) is a common chronic liver metabolic disease worldwide. Up to 70%–80% of patients with NAFLD were obese, especially abdominal obesity. Many indicators of abdominal obesity have been reported, including waist circumference (WC), visceral obesity index (VAI), lipid accumulation (LAP), and Chinese VAI (CVAI). However, few studies investigated the associations between these indices with NAFLD. This present study aims to explore the associations between abdominal obesity indices with NAFLD. A total of 7,238 participants were involved in the cross-sectional study, and 1,584 participants were included in the longitudinal study from Jidong communities. NAFLD was assessed by abdominal ultrasonography. The trajectory of WC, VAI, LAP, and CVAI during 2013–2016 was identified by a group-based trajectory model. The logistic regression and Cox proportional hazards models analyzed the correlations and causality between abdominal obesity indices with NAFLD. In this study, the prevalence and incidence of NAFLD are approximately 44% and 26%, respectively. In the cross-sectional study, WC, VAI, LAP, and CVAI are associated with NAFLD. After adjustment for potential confounders, the moderate-rising and high-rising groups of CVAI had the highest risk of NAFLD in longitudinal analysis (hazard ratio (HR): 3.903, 95%CI: 2.434–6.259; HR: 5.694 95%CI: 3.098–10.464, respectively). Receiving operating characteristic curves show that CVAI has the best diagnostic value for NAFLD (area under the curve (AUC) = 0.868). CVAI is independently associated with the risk of NAFLD and may also have an important value to the diagnosis of NAFLD.

## Introduction

Obesity is a significant public health issue associated with increased morbidity and disease burden worldwide. In 2016, over 1.9 billion people worldwide were overweight, and over 650 million were obese (1). Obesity is becoming increasingly prevalent with multiple adverse consequences. Obesity is linked to many metabolic diseases and is particularly correlated with non-alcoholic fatty liver disease (NAFLD) in some epidemiological studies (2, 3).

The national prevalence of NAFLD was estimated to be 29.2% in China, which has imposed substantial health and economic burden on patients and society (4). NAFLD is a common chronic liver metabolic disease characterized by excessive fat accumulation in the liver, which affects over 30% of the adult and 70%–80% of the obese population (5). The clinical–histologic phenotype of NAFLD ranges from fatty liver to non-alcoholic steatohepatitis (NASH) to cirrhosis (6).

In recent years, the incidence of NAFLD has been increasing worldwide, which is driven by the obesity epidemic (7). Notably, NAFLD also occurs in non-obese individuals with normal body mass index (BMI). Moreover, BMI, a remarkably heterogeneous parameter, is used as a surrogate indicator of body fat content but cannot reflect body fat distribution (8). Recent studies indicated that adipose tissue distribution instead of the actual amount of body fat might play a more crucial role in the progression of metabolic diseases or all-cause mortality (9–11).

The development of imaging technologies such as CT and MRI has made it possible to explore the field of adipose tissue distribution (12). The proportion of abdominal adipose tissue may be a related factor of obesity-related NAFLD. Because imaging examinations have the disadvantages of time-consuming, expensive, and radiation exposure, many indicators for assessing abdominal obesity have been established (13). Waist circumference (WC) is regarded as an important indicator for central obesity recommended by the WHO (14). Visceral obesity index (VAI), lipid accumulation (LAP), and Chinese VAI (CVAI) for assessing visceral obesity were calculated by fundamental indicators such as age, WC, BMI, high-density lipoprotein (HDL), and triglycerides (TG) (15, 16). In addition, CVAI was a newly established index to evaluate visceral fat obesity. To our knowledge, the evidence for the associations of WC, VAI, LAP, and CVAI with NAFLD is still limited. Furthermore, the relationship between these indicators and metabolic abnormalities such as type 2 diabetes and metabolic syndrome has been discovered; findings implied the predictive value of these indices for metabolic abnormalities (17, 18). Notably, CVAI was a novel VAI based on Chinese adults, which has been shown to be well correlated to the visceral fat area and homeostasis model assessment for insulin resistance and was considered as a good marker of cardiometabolic risk and incident hypertension (16, 19, 20).

This large-scale community-based study contained cross-sectional and longitudinal data in North China. We aimed to investigate the relationships between abdominal obesity indices, including WC, VAI, LAP, and CVAI, with the prevalence of NAFLD. Our findings may provide important implications for the early detection primary prevention of NAFLD.

## Methods

### Study Design and Participants

**Figure 1:**
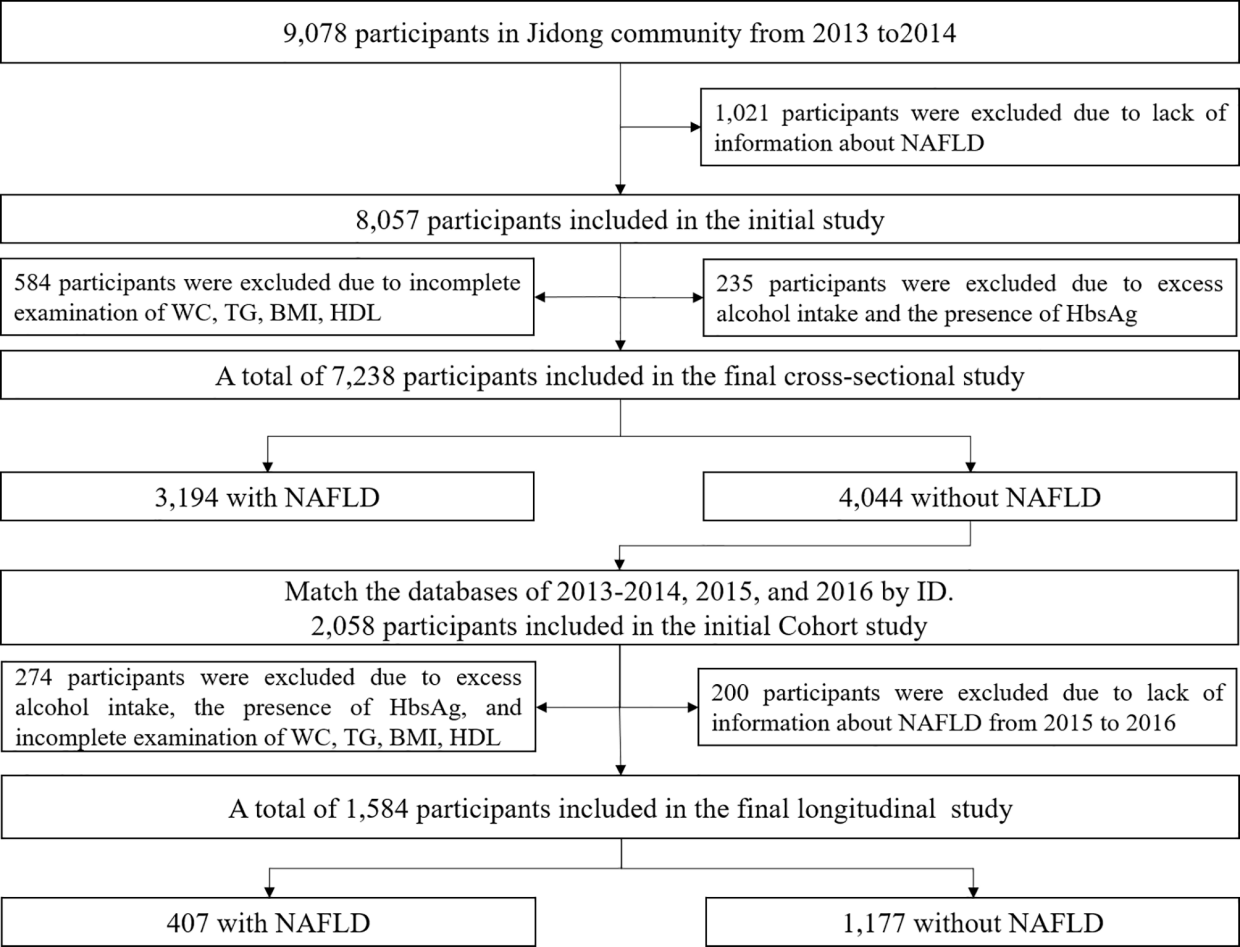
Flowchart of this study. NAFLD, non-alcoholic fatty liver disease; BMI, body mass index; HDL, high-density lipoprotein; TG, total cholesterol; WC, waist circumference.

This study was designed to investigate the association between abdominal obesity indices with NAFLD among Chinese adults. The participants in this study were recruited from Jidong communities in Tangshan City (Hebei Province, North China). From 2013 to 2014, 9,078 participants received examinations, and participants were excluded due to lack of information about NAFLD (n = 1021), excess alcohol intake (one drink or more daily among women and two drinks or more daily among men), the presence of the hepatitis B surface antigen (HBsAg) (n = 235), and missing the data of WC, TG, BMI, or HDL (n = 584). In total, 7,238 participants were involved in the cross-sectional study. In the longitudinal study, by matching ID numbers from the datasets of 2013–2014, 2015, and 2016, the information of 2,058 participants without NAFLD was acquired. A total of 1,584 participants were included in the final longitudinal study, and 474 participants were excluded for missing the data of NAFLD or WC, TG, BMI, or HDL, or excess alcohol intake, or the presence of the HBsAg from 2015 to 2016 (**Figure 1**).

The study was conducted in accordance with the guiding principles of the Declaration of Helsinki and approved by the Medical Ethics Committee, Staff Hospital, Jidong Oilfield Branch, China National Petroleum Corporation (approval No. 2013 YILUNZI 1). Written informed consent was obtained from all participants.

### Assessment of Non-Alcoholic Fatty Liver Disease

After exclusion of participants with excessive drinking and other liver diseases, participants with two or more of the following abnormal characteristics were considered to have NAFLD: 1) diffusely increased echogenicity of the liver relative to the kidney; 2) ultrasound beam attenuation; and 3) poor visualization of intrahepatic structures. NAFLD was diagnosed in accordance with the Asia‐Pacific Working Party on NAFLD and the Chinese Association for the Study of Liver Disease criteria (21, 22). The abdominal ultrasonography examination was performed using a high‐resolution B‐mode topographical ultrasound system with a 3.5-MHz probe (ACUSON X300, Siemens, Munich, Germany) by skilled sonographers following a standardized protocol.

### Assessment of Abdominal Obesity Indices

WC was measured twice by the technician at the level of the umbilicus, and the average value was used to determine the WC of each participant. Fundamental indicators calculated the VAI, LAP, and CVAI, and the specific calculation process as follows:


$$\text{Male}:\text{VAI}=\text{WC (cm)/[}39.68+1.88\times {\text{BMI (kg/m}}^{2}\text{)]}\times [\text{TG (mmol/L)}/1.03]\times [1.31\text{/HDL (mmol/L)}]\text{LAP}=[\text{WC (cm)}-65]\times \text{TG }(\text{mmol/L})$$

$$\text{CVAI}=-267.93+0.68\times \text{age (years)}+0.03\times {\text{BMI (kg/m}}^{2})+4.00\times \text{WC (cm)}+22.00\times \text{Lg TG (mmol/L)}-16.32\times \text{HDL (mmol/L)}$$

$$\text{Female}:\text{VAI}=\text{WC (cm)}/[36.58+1.89\times {\text{BMI (kg/m}}^{2})]\times [\text{TG (mmol/L)/}0.81]\times [1.52\text{/HDL (mmol/L)}]$$

LAP=[WC (cm)−58]×TG (mmol/L)

$$\text{CVAI}=-187.32+1.71\times \text{age (years)}+4.32\times {\text{BMI (kg/m}}^{2})+1.12\times \text{WC (cm)}+39.76\times \text{Lg TG (mmol/L)}-11.66\times \text{HDL (mmol/L)}$$

### Assessment of Covariates

All participants completed a standardized questionnaire, clinical examinations, and laboratory tests. The questionnaire covered demographic and lifestyle information collected by the researchers who had received professional training. According to self-reported information, the information on smoking habits (current smoker or not) and drinking habits (current drinker or not, drinking quantity) was obtained. Education level was categorized as “illiteracy or primary school”, “middle school”, or “college and above”. Participants’ average monthly earnings were classified as “≤¥3,000”, “¥3,001–5,000”, or “>¥5,000”.

Clinical examinations were performed by experienced nurses and included measurements of height, weight, WC, and blood pressure. BMI was calculated by weight (kg)/height (m)^2^. Systolic blood pressure (SBP) and diastolic blood pressure (DBP) were taken twice in a sitting position and averaged. Blood samples were collected using venipuncture after an overnight fast. Fasting plasma glucose (FPG), TG, total cholesterol (TC), HDL, and low-density lipoprotein (LDL) were performed with a Hitachi 747 auto-analyzer (Hitachi, Tokyo, Japan). Hypertension was defined as SBP ≥ 140 mmHg and/or DBP ≥ 90 mmHg or hypertension history or currently taking medication for hypertension. Diabetes was defined by any one of the following: FPG level ≥7.0 mmol/L, use of diabetes medication, or diabetes history.

### Statistical Analyses

Statistical analyses were performed using SAS, version 9.4 (SAS Institute Inc., Cary, NC, USA), and the significance level was set as *p*-value (two-sided) <0.05. The normality distribution of continuous variables was assessed by the Kolmogorov–Smirnov test. Continuous variables were expressed as the median with interquartile range (IQR; 25%–75%), and categorical variables were presented as frequencies and percentages in cross-sectional or longitudinal studies. Continuous and categorical variables were compared using the Kruskal–Wallis H tests and chi-square tests, respectively. Multicollinearity was assessed by the variance inflation factor (VIF). Logistic regression analysis was used to analyze the relationship between WC, VAI, LAP, and CVAI with NAFLD by calculating the odds ratios (ORs) and 95%CI in a cross-sectional study.

Longitudinal analyses by the group-based trajectory identified groups of participants by changes of abdominal obesity index from 2013 to 2016, and model fit was assessed using the Bayesian information criterion (BIC). The focus was on this trajectory model because it is easy to achieve using “Proc Traj,” a free downloadable add-on package to base SAS (http://www.andrew.cmu.edu/user/bjones/index.htm), and because it was shown to be superior for identifying underlying longitudinal trajectories (23). In the group-based trajectory models, several regression models are estimated simultaneously by maximizing a likelihood that combines the information from all models. In addition, the Cox proportional hazards model estimated the hazard ratios (HRs) and 95%CI to assess NAFLD between different trajectory groups. To assess the accuracy of the estimation, a sensitivity analysis was performed to further adjust for continuous abdominal obesity indicators in 2013 and 2016, one at a time. The diagnostic ability of abdominal obesity indices including WC, VAI, LAP, and CVAI for NAFLD was analyzed by receiver operating characteristics (ROC) curve analysis. Meanwhile, the abdominal obesity indices were used in the dose–response diagram as horizontal coordinates and the corresponding OR value as vertical coordinates.

## Results

### Descriptive Characteristics of the Participants at Baseline and Follow-Up

**Table 1** describes the basic characteristics of participants according to NAFLD status at baseline and follow-up. Overall, 7,238 participants were involved in the cross-sectional analysis; 44.1% were participants with NAFLD, of whom 60.6% were male. Among participants, the median age between with and without NAFLD was 45.6, 37.4, respectively. A total of 1,584 participants were involved in the longitudinal analysis, and 25.7% of them developed NAFLD during the follow-up. The median duration of follow-up was 32 months (range: 10–36). Among the NAFLD group, the median age was 44 years; about 46.2% were male. Significant differences were observed in age, sex, the habit of smoking/drinking, income, and the prevalence of diabetes and hypertension between participants with/without NAFLD in two analysis sections. Neither cross-sectional nor longitudinal data of participants with NAFLD were more likely to have higher BMI, SBP, DBP, fasting blood glucose (FBG), LDL, TG, TC, WC, VAI, LAP, and CVAI and lower levels of HDL (*p* < 0.05).

**Table 1:** Characteristics of participants according to NAFLD status among cross-sectional and longitudinal datasets.

	Cross-sectional study			*p*-Value	Longitudinal study^b^			*p*-Value
	Total	Without NAFLD	With NAFLD		Total	Without NAFLD	With NAFLD
Variables	7,238	4,044 (55.9)	3,194 (44.1)		1,584	1,177 (74.3)	407 (25.7)	
Age, year^a^	41.6 (30.9, 53.3)	37.4 (29.5, 49.5)	45.6 (33.3, 58.0)	<0.001	38.9 (30.7, 52.4)	37.7 (30.7, 51.1)	44.1 (31.6, 56.6)	<0.001
Male, n (%)	3,269 (45.2)	1,333 (33.0)	1,936 (60.6)	<0.001	559 (35.3)	371 (31.5)	188 (46.2)	<0.001
Current smoking, n (%)	1,530 (21.1)	570 (14.1)	960 (30.1)	<0.001	216 (13.6)	138 (11.7)	78 (19.2)	<0.001
Current drinking, n (%)	1,600 (22.1)	629 (15.6)	971 (30.4)	<0.001	265 (16.7)	178 (15.1)	87 (21.4)	0.004
Income ¥/month, n (%)				0.003				0.003
≤3,000	2,846 (39.9)	1,507 (37.9)	1,339 (42.5)		623 (40.0)	436 (37.8)	187 (46.5)	
3,001–5,000	3,783 (53.1)	2,201 (55.3)	1,582 (50.3)		838 (53.9)	651 (56.4)	187 (46.5)	
>5,000	498 (7.0)	271 (6.8)	227 (7.2)		95 (6.1)	67 (5.8)	28 (7.0)	
Education level, n (%)				<0.001				0.080
Illiteracy/primary	292 (4.03)	133 (3.3)	159 (5.0)		64 (4.0)	48 (4.1)	16 (3.9)	
Middle school	2,655 (36.7)	1,305 (32.3)	1,350 (42.3)		543 (34.3)	385 (32.7)	158 (38.8)	
College or above	4,291 (59.3)	2,606 (64.4)	1,685 (52.8)		977 (61.7)	744 (63.2)	233 (57.3)	
BMI,^a^ kg/m^2^	24.1 (21.7, 26.6)	22.3 (20.5, 24.2)	26.4 (24.6, 28.7)	<0.001	22.6 (20.8, 24.4)	22.0 (20.4, 23.7)	24.6 (22.9, 26.1)	<0.001
SBP, mmHg^a^	123.0 (113.0, 136.0)	118.0 (108.0, 129.0)	130.0 (120.0, 144.0)	<0.001	119.7 (111.7, 129.3)	118.0 (110.3, 127.0)	126.3 (117.3, 136.0)	<0.001
DBP, mmHg^a^	79.0 (71.0, 88.0)	75.0 (68.0, 83.0)	84.0 (77.0, 94.0)	<0.001	75.3 (70.0, 82.7)	74.3 (69.0, 80.7)	79.7 (73.386.0)	<0.001
FBG, mmol/L^a^	5.0 (4.7, 5.4)	4.9 (4.6, 5.2)	5.2 (4.8, 5.7)	<0.001	5.2 (4.9, 5.4)	5.1 (4.9, 5.4)	5.3 (5.0, 5.6)	<0.001
HDL, mmol/L^a^	1.2 (1.0, 1.4)	1.3 (1.1, 1.5)	1.1 (0.9, 1.2)	<0.001	1.3 (1.1, 1.4)	1.3 (1.1, 1.5)	1.2 (1.0, 1.3)	<0.001
LDL, mmol/L^a^	2.4 (2.0, 2.9)	2.3 (1.9, 2.7)	2.6 (2.3, 3.0)	<0.001	2.6 (2.2, 3.0)	2.5 (2.1, 2.9)	2.8 (2.4, 3.2)	<0.001
TG, mmol/L^a^	1.2 (0.8, 1.8)	1.0 (0.7, 1.3)	1.7 (1.2, 2.4)	<0.001	1.1 (0.8, 1.4)	1.0 (0.8, 1.3)	1.4 (1.1, 2.0)	<0.001
TC, mmol/L^a^	4.4 (3.8, 5.0)	4.2 (3.7, 4.8)	4.6 (4.1, 5.2)	<0.001	4.5 (4.0, 5.0)	4.4 (3.9, 4.9)	4.7 (4.1, 5.3)	<0.001
Hypertension, n (%)	2,210 (30.5)	693 (17.1)	1,517 (47.5)	<0.001	260 (16.4)	160 (13.6)	100 (24.6)	<0.001
Diabetes, n (%)	483 (6.7)	136 (3.4)	347 (10.9)	<0.001	50 (3.2)	27 (2.3)	23 (5.7)	0.008
WC, cm^a^	85.0 (78.0, 92.0)	80.0 (74.0, 86.0)	90.0 (85.0, 96.0)	<0.001	80.3 (74.7, 86.0)	78.3 (73.3, 84.0)	85.8 (81.3, 90.3)	<0.001
VAI ^a^	1.6 (1.0, 2.6)	1.2 (0.9, 1.8)	2.4 (1.5, 3.7)	<0.001	1.4 (1.0, 2.1)	1.3 (1.0, 1.8)	2.0 (1.4, 3.0)	<0.001
LAP^a^	27.9 (15.3, 49.8)	17.8 (11.1, 29.5)	47.0 (30.6, 74.0)	<0.001	21.5 (13.4, 33.5)	17.8 (11.9, 27.0)	35.4 (23.7, 50.9)	<0.001
CVAI^a^	84.4 (46.1, 117.7)	55.8 (27.0, 86.0)	114.7 (91.2, 138.5)	<0.001	64.3 (34.6, 93.5)	52.4 (27.2, 82.0)	92.4 (70.0, 113.1)	<0.001

NAFLD, non-alcoholic fatty liver disease; BMI, body mass index; SBP, systolic blood pressure; DBP, diastolic blood pressure; FBG, fasting blood glucose; HDL, high-density lipoprotein; LDL, low-density lipoprotein; TG, total cholesterol; TC, total cholesterol; WC, waist circumference; VAI, visceral obesity index; LAP, lipid accumulation; CVAI, Chinese visceral obesity index; IQR, interquartile range.

a Data are median (IQR).

b Average values based on measurements in 2013–2014, 2015, and 2016.

### Correlation Between Abdominal Obesity Indices With Non-Alcoholic Fatty Liver Disease in the Cross-Sectional Analysis

**Figure 2:**
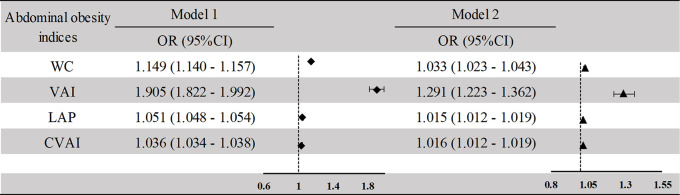
Association between the abdominal obesity indices and NAFLD in the cross-sectional study. Model 1: unadjusted model. Model 2: adjusted for age, sex, current smoking, current drinking, income, education level, BMI, SBP, DBP, FBG, HDL, TC, hypertension, diabetes. NAFLD, non-alcoholic fatty liver disease; BMI, body mass index; SBP, systolic blood pressure; DBP, diastolic blood pressure; FBG, fasting blood glucose; HDL, high-density lipoprotein; TC, total cholesterol; WC, waist circumference; VAI, visceral obesity index; LAP, lipid accumulation; CVAI, Chinese visceral obesity index.

**Figure 2** indicates that increased WC, VAI, LAP, and CVAI were significantly associated with a greater prevalence of NAFLD in cross-sectional analysis. The ORs and 95%CIs of NAFLD for WC, VAI, LAP, and CVAI in the adjusted model were 1.033 (1.023–1.044), 1.291 (1.223–1.362), 1.015 (1.012–1.019), and 1.016 (1.013–1.019), respectively. The adjusted variables were age, sex, education level, smoking or drinking habits, hypertension, diabetes SBP, DBP, FBG, TC, and HDL, removing the variables LDL and TG (VIF > 10).

### The Trajectory of Abdominal Obesity Indices From 2013 to 2016

**Figure 3:**
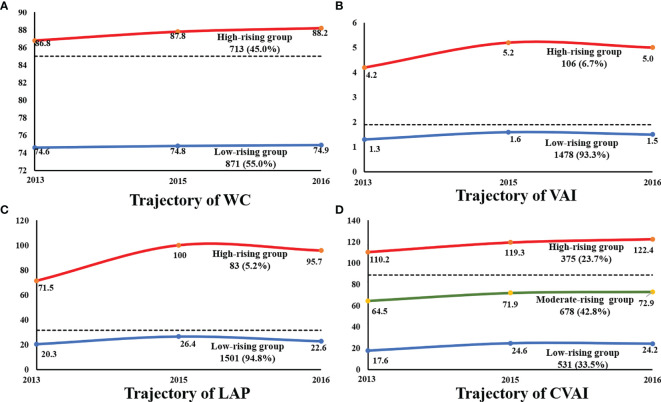
The trajectory of abdominal obesity indices during 2013-2016. **(A)** The trajectory of WC. **(B)** The trajectory of VAI. **(C)** The trajectory of LAP. **(D)** The trajectory of CVAI. WC, waist circumference; VAI, visceral obesity index; LAP, lipid accumulation; CVAI, Chinese visceral obesity index.

We categorized the study population based on two observed discrete trajectories of WC, VAI, and LAP from 2013 to 2016, labeled as low-rising (WC ranged from 74.6 to 74.9, n = 871, 55.0%; VAI ranged from 1.3 to 1.5, n = 1478, 93.3%; LAP ranged from 20.3 to 22.6, n = 1501, 94.8%) and high-rising (WC ranged from 86.8 to 88.2, n = 713, 45.0%; VAI ranged from 4.2 to 5.0, n = 106, 6.7%; LAP ranged from 71.5 to 95.7, n = 83, 5.2%; n = 375, 23.7%). Participants of the longitudinal study were clustered into three groups according to the trajectory of changing patterns in CVAI over time. According to the initial value, all the change trajectories are upward trends and are divided into low-rising, medium-rising, and high-rising. In the low-rising group, the CVAI remained with a range of 17.6–24.2. The CVAI remained at a moderate level in the moderate-rising group and rose slightly (64.5 to 72.9). The initial level of CVAI was as high as 110.2, and CVAI increased to 122.4 after follow-up, which was defined as the high-rising group (**Figure 3**) . Characteristics of participants of the longitudinal analysis, grouped by the different abdominal obesity indices, are shown in **Supplementary Tables S1** and **S2**.

### Associations Between Abdominal Obesity Indices With Non-Alcoholic Fatty Liver Disease in the Longitudinal Analysis

**Figure 4:**
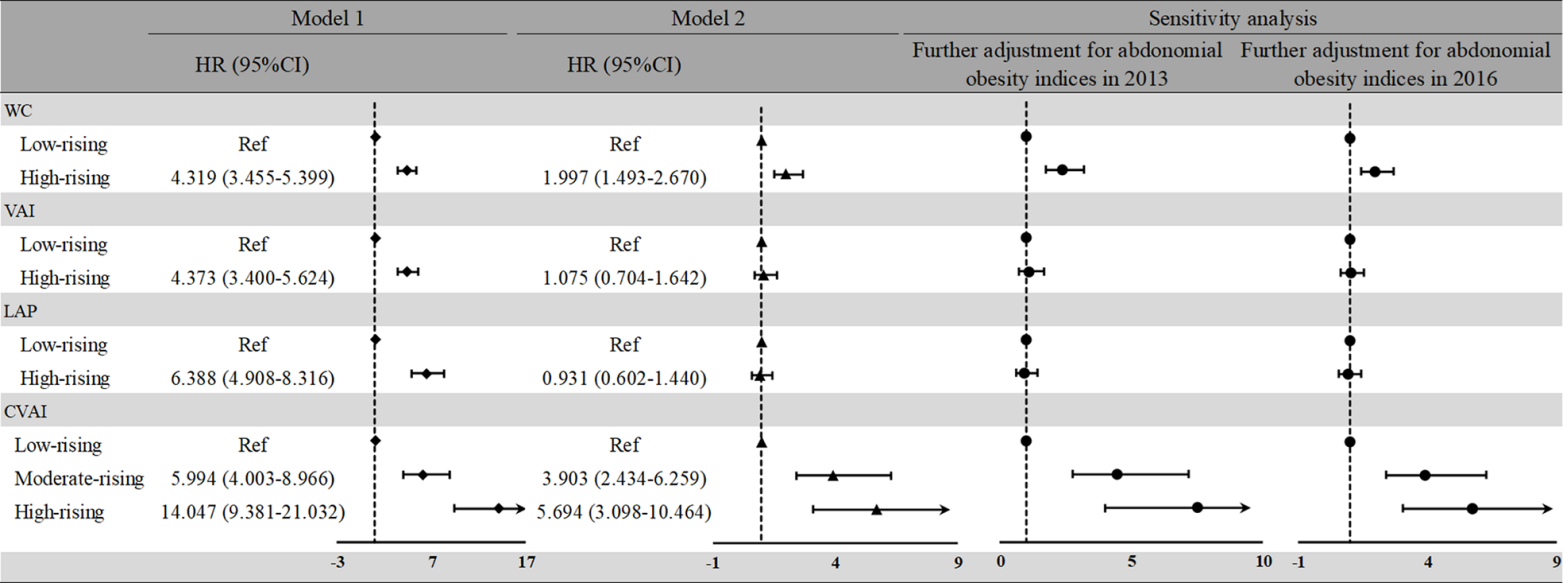
HRs and 95%CIs for risk of NAFLD according to the abdominal obesity indices trajectory patterns during 2013 to 2016. Model 1: unadjusted model. Model 2: adjusted for age, sex, current smoking, current drinking, income, education level, and the average measurement of BMI, SBP, DBP, FBG, HDL, TC, TG, hypertension, and diabetes. NAFLD, non-alcoholic fatty liver disease; BMI, body mass index; SBP, systolic blood pressure; DBP, diastolic blood pressure; FBG, fasting blood glucose; HDL, high-density lipoprotein; TG, total cholesterol; TC, total cholesterol; WC, waist circumference; VAI, visceral obesity index; LAP, lipid accumulation; CVAI, Chinese visceral obesity index; HR, hazard ratio.

The high-rising CVAI pattern experienced the highest future risk of developing NAFLD among all abdominal obesity indices patterns (**Figure 4**). Relative to WC, VAI, and LAP of the low-rising group, adjusted high-rising group’s HRs were 1.997 (95%CI: 1.493–2.670) for WC, 1.075 (95%CI: 0.704–1.642) for VAI, and 0.931 (95%CI: 0.602–1.440) for LAP, after adjustment for potential confounders (excluding LDL, VIF > 10). The moderate-rising and high-rising CVAI groups both had a higher risk of NAFLD (HR: 5.994, 95%CI: 4.003–8.966; HR: 14.047, 95%CI: 9.381–21.032, respectively) in the crude model, compared with the low-rising group. Similar associations between the moderate-rising and high-rising CVAI groups with NAFLD were observed in the adjusted model (HR: 3.903, 95%CI: 2.434–6.259; HR: 5.694, 95%CI: 3.098–10.464, respectively). After two additional sensitivity analyses, the trajectory groups of CVAI still had the highest positive association with NAFLD.

### Diagnostic Ability of Abdominal Obesity Indices for Non-Alcoholic Fatty Liver Disease

**Figure 5:**
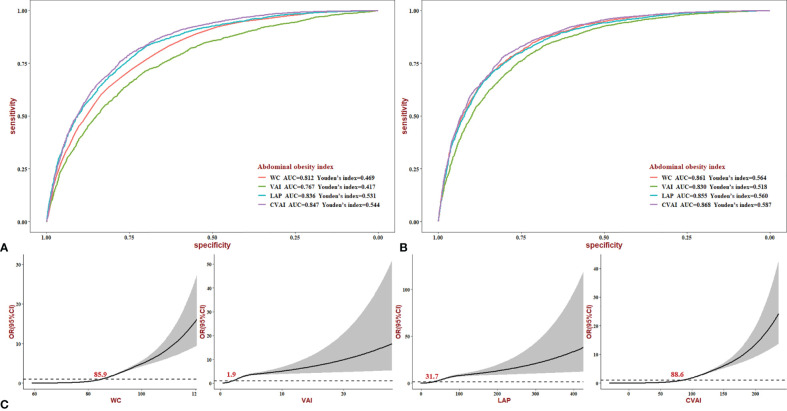
ROC and dose–response curves of abdominal obesity indices for NAFLD. **(A)** Crude ROC curves. **(B)** Adjusted ROC curves (age, sex, current smoking, current drinking, income, education level, hypertension, diabetes, BMI, SBP, DBP, FBG, HDL, TC, hypertension, and diabetes.). **(C)** Dose–response relationship between the risk of NAFLD and changes in WC, VAI, LAP, and CVAI level. NAFLD, non-alcoholic fatty liver disease; BMI, body mass index; SBP, systolic blood pressure; DBP, diastolic blood pressure; FBG, fasting blood glucose; HDL, high-density lipoprotein; TC, total cholesterol; WC, waist circumference; VAI, visceral obesity index; LAP, lipid accumulation; CVAI, Chinese visceral obesity index.

In the crude model, the area under the ROC curve (AUC) of WC, VAI, LAP, and CVAI for NAFLD was 0.812, 0.767, 0.836, and 0.847 (*p* < 0.05) (**Figure 5A**). Compared with WC, VAI, and LAP, CVAI had the biggest AUC, with Youden’s index being 0.544. The adjustment slightly increased the AUC, with CVAI still with the best diagnostic value (AUC = 0.868, *p* < 0.05, Youden’s index = 0.587) (**Figure 5B**). In addition, we stratified the analyses according to sex, as shown in **Figure S2**. In women, CVAI still has reliable diagnostic power (AUC = 0.875, *p* < 0.05, Youden’s index = 0.592). The dose–response relationship between abdominal obesity indices and NAFLD risk was analyzed using the restricted cubic spline regression model (**Figure 5C**). The risk of NAFLD increased with increasing WC, VAI, and LAP when the indices were higher than 85.9, 1.9, and 31.7, respectively. Notably, CVAI, which has the biggest diagnostic value, could increase NAFLD risk when higher than 88.6.

## Discussion

This study assessed the associations of the multiple abdominal obesity indicators with NAFLD risks among Chinese people. We identified that CVAI had the strongest associations with the incidence of NAFLD among these abdominal obesity indices in the longitudinal study, and all the associations were independent of BMI. CVAI might be a useful tool in daily clinical practice for the assessment of NAFLD.

The influence of abdominal fat accumulation on NAFLD development has been reported previously (24, 25), and the conclusion was also corroborated in our research. CVAI, correlated with visceral adipose tissue (VAT), is better to predict obesity-related metabolic disorders than WC, LAP, and BMI (**Supplementary Table S3**). The reason may be WC and LAP only reflect the amount of abdominal fat without distinguishing the amount of visceral and subcutaneous fat, and BMI could not reflect regional fat distribution, while CVAI reflects the amount of visceral fat (26, 27). VAT could release free fatty acids and enter the portal vein to form TG in the liver to induce hepatocyte inflammation, which was considered to be the most important factor contributing to liver damage in NAFLD (28–30). Our data indicated that the CVAI, among the common indices of abdominal obesity assessment, would be a better clinical abdominal obesity indicator for NAFLD risk.

The association between some abdominal obesity indicators with NAFLD has been described in the past. VAI was considered as a surrogate marker of hepatic steatosis for detecting NAFLD in the case–control study (31). This finding was consistent with the result of our cross-sectional study, but the correlation could not prove causality, and cross-sectional results need to be confirmed prospectively. However, our longitudinal results disproved the prior conclusion and demonstrated the value of CVAI in the process of NAFLD.

Previous studies reported that CVAI was superior to the traditional estimates for the diagnosis of metabolic diseases (16, 32), which is similar to the finding of our research. Our results indicated that visceral adiposity estimated by CVAI had a better prediction of incident NAFLD. The AUC and Youden’s index of CVAI for the diagnosis of NAFLD were the largest among WC, VAI, LAP, and CVAI in Chinese adults. This may be related to the good correlation between CVAI with visceral fat mass in Chinese adults, which has been confirmed in another study (26). At the same time, CVAI was composed of the components of metabolic syndrome, which were easily available in clinical practice (33, 34). Thus, CVAI was a reliable clinical abdominal obesity indicator for NAFLD risk when biospecimen data were available.

This study has several limitations. Due to the lack of information on the other type of liver diseases, such as autoimmune hepatitis (AIH) and cholestatic disease, the participants with these medications were not excluded, which is one of the limitations of our study. In addition, the current study did not evaluate the visceral fat function parameters and did not directly measure the visceral fat function area but only referred to the description of the abdominal obesity index in other studies. Therefore, we could not further verify the relationship between CVAI and VAT. Our study did not consider some potential impact of unmeasured confounders on the main results, such as changes in diet, medication, or comorbidity; thus, our results should be interpreted with caution. The key strength of our research includes cross-sectional and longitudinal data collected from a community with standardized measurement. To the best of our knowledge, it is the first study to detect the various associations of obesity phenotype indices with NAFLD and predict the power of abdominal obesity indices.

## Conclusion

In conclusion, the present study indicated that CVAI was the strongest and independent risk factor for NAFLD among the abdominal obesity indices. Compared to WC, VAI, and LAP, CVAI demonstrates the higher diagnosis power for NAFLD based on the ROC curve, especially in women. Overall, the CVAI may be a practical and straightforward approach for predicting NAFLD, and individuals with high CVAI should receive additional screening and preventive interventions for NAFLD.

## Data Availability Statement

The raw data supporting the conclusions of this article will be made available by the authors, without undue reservation.

## Ethics Statement

The studies involving human participants were reviewed and approved by Medical Ethics Committee, Staff Hospital, Jidong Oilfield Branch, China National Petroleum Corporation (approval No. 2013 YILUNZI 1). The patients/participants provided their written informed consent to participate in this study.

## Author Contributions

All authors equally contributed to the present study.

## Funding

This project was supported by the Doctoral supervisor matching funds (grant no. 6010220052), the Research Development Fund of The Second Hospital of Shandong University (grant no. 11681808), and Jinan clinical medical science and technology innovation plan (grant no. 202019194).

## Conflict of Interest

The authors declare that the research was conducted in the absence of any commercial or financial relationships that could be construed as a potential conflict of interest.

## Publisher’s Note

All claims expressed in this article are solely those of the authors and do not necessarily represent those of their affiliated organizations, or those of the publisher, the editors and the reviewers. Any product that may be evaluated in this article, or claim that may be made by its manufacturer, is not guaranteed or endorsed by the publisher.
