# Supplementary figures and images for: Associations Between Abdominal Obesity Indices and Nonalcoholic Fatty Liver Disease: Chinese Visceral Adiposity Index
Source: Front Endocrinol (Lausanne). 2022 Mar 10;13:831960. doi: 10.3389/fendo.2022.831960 (PMC8960385; doi:10.3389/fendo.2022.831960)

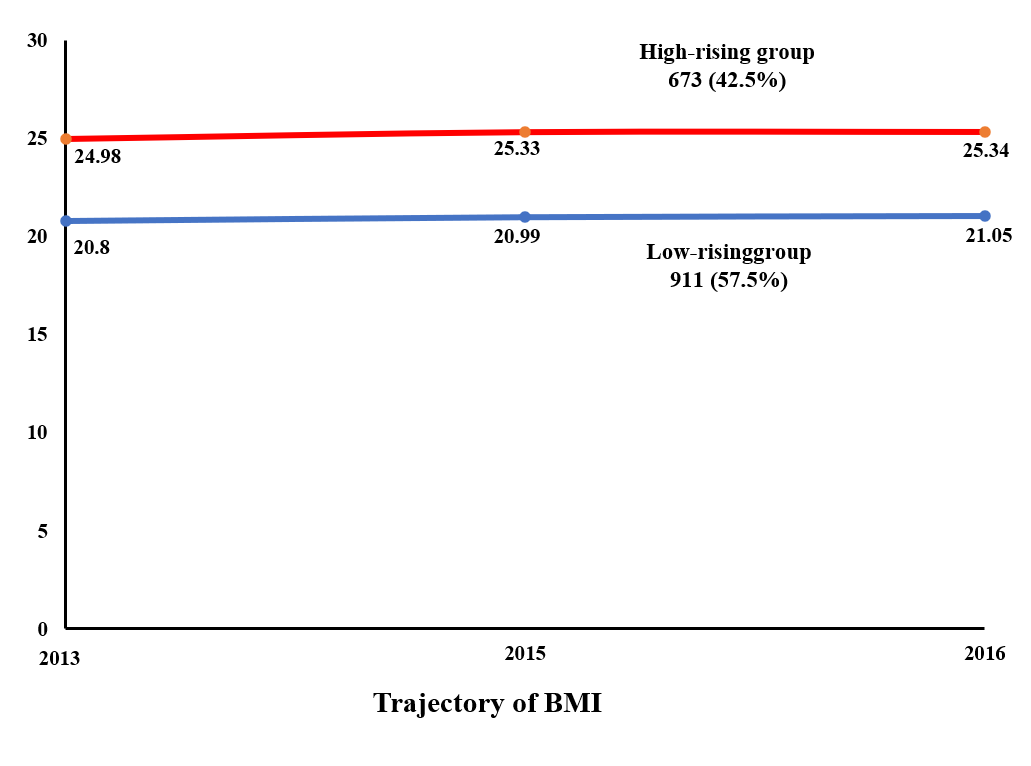

Supplement: Supplementary file 1 [file Image_1.tif]

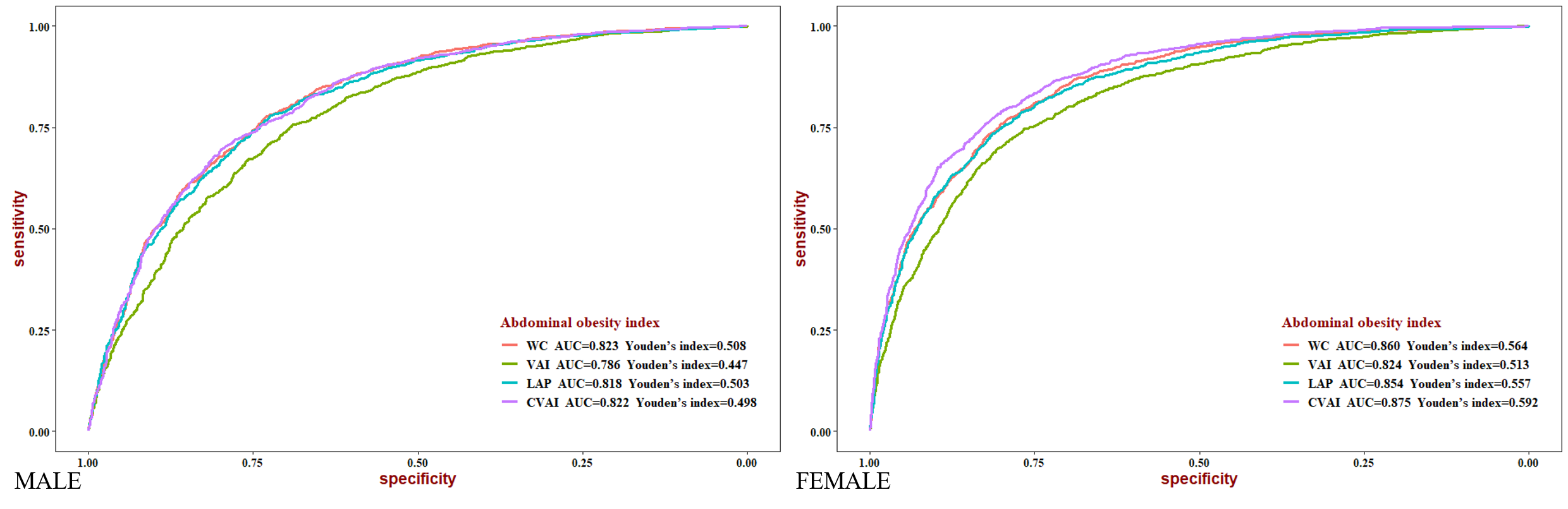

Supplement: Supplementary file 2 [file Image_2.tif]
