# Supplementary material for: Associations Between Abdominal Obesity Indices and Nonalcoholic Fatty Liver Disease: Chinese Visceral Adiposity Index
Source: Front Endocrinol (Lausanne). 2022 Mar 10;13:831960. doi: 10.3389/fendo.2022.831960 (PMC8960385; doi:10.3389/fendo.2022.831960)
Supplement: Supplementary file 3 [file Table_1.docx]

Table S1. Basic characteristics according to the WC and VAI trajectory patterns from 2013 to 2016.

|  | Total | WC trajectory groups | | |  | VAI trajectory groups | | |
| --- | --- | --- | --- | --- | --- | --- | --- | --- |
|  |  | Low-rising | High-rising | *P* value |  | Low-rising | High-rising | *P* value |
| Variables n (%) | 1584 | 871 (55.0) | 713 (45.0) |  |  | 1478 (93.3) | 106 (6.7) |  |
| NAFLD n (%) | 407 (25.7) | 104 (11.9) | 303 (42.5) | <0.001 |  | 332 (22.5) | 75 (70.8) | <0.001 |
| Age,year ^ab^ | 38.9 (30.7, 52.4) | 35.6 (39.9, 46.7) | 46.7 (32.1, 59.6) | <0.001 |  | 38.7 (30.7, 52.1) | 43.9 (31.7, 53.6) | 0.242 |
| Male, n (%) | 559 (35.3) | 151 (17.3) | 408 (57.2) | <0.001 |  | 519 (35.1) | 40 (37.7) | 0.586 |
| Current smoking, n (%) | 216 (13.6) | 57 (6.5) | 159 (22.3) | <0.001 |  | 193 (13.1) | 23 (21.7) | 0.014 |
| Current drinking, n (%) | 265 (16.7) | 79 (9.1) | 186 (26.1) | <0.001 |  | 245 (16.6) | 20 (18.9) | 0.542 |
| Income ￥/month, n (%) |  |  |  | <0.001 |  |  |  | 0.174 |
| ≤3000 | 623 (40.0) | 292 (34.2) | 331 (47.2) |  |  | 581 (40.0) | 42 (40.4) |  |
| 3001-5000 | 838 (53.9) | 505 (59.1) | 333 (47.4) |  |  | 778 (53.6) | 60 (57.7) |  |
| ＞5000 | 95 (6.1) | 57 (6.7) | 38 (5.4) |  |  | 93 (6.4) | 2 (1.9) |  |
| Education level, n (%) |  |  |  | <0.001 |  |  |  | 0.267 |
| Illiteracy/primary | 64 (4.0) | 19 (2.2) | 45 (6.3) |  |  | 60 (4.1) | 4 (3.8) |  |
| Middle school | 543 (34.3) | 254 (29.2) | 289 (40.5) |  |  | 499 (33.8) | 44 (41.5) |  |
| College or above | 977 (61.7) | 598 (68.7) | 379 (53.2) |  |  | 919 (62.2) | 58 (54.7) |  |
| BMI, kg/m^2 ab^ | 22.6 (20.8, 24.4) | 21.2 (19.8, 22.5) | 24.5 (23.2, 25.9) | <0.001 |  | 22.5 (20.8, 24.3) | 24.4 (22.7, 26.2) | <0.001 |
| SBP, mmHg ^ab^ | 119.7 (111.7, 129.3) | 115.3 (108.3, 123.7) | 126.3 (117.3, 135.7) | <0.001 |  | 119.3 (111.3, 129.0) | 125.2 (117.3, 139.0) | 0.001 |
| DBP, mmHg ^ab^ | 75.3 (70.0, 82.7) | 72.7 (66.0, 79.0) | 79.3 (73.3, 86.3) | <0.001 |  | 75.3 (69.7, 82.3) | 79.5 (73.0, 86.7) | <0.001 |
| FBG, mmol/L ^ab^ | 5.2 (4.9, 5.4) | 5.1 (4.9, 5.3) | 5.3 (5.0, 5.6) | <0.001 |  | 5.2 (4.9, 5.4) | 5.4 (5.1, 5.6) | <0.001 |
| HDL, mmol/L ^ab^ | 1.3 (1.1, 1.4) | 1.3 (1.2, 1.5) | 1.2 (1.0, 1.4) | <0.001 |  | 1.3 (1.1, 1.5) | 1.0 (0.9, 1.1) | <0.001 |
| LDL, mmol/L  ^ab^ | 2.6 (2.2, 3.0) | 2.4 (2.0, 2.8) | 2.7 (2.4, 3.1) | <0.001 |  | 2.3 (2.2, 2.9) | 2.8 (2.4, 3.2) | <0.001 |
| TG, mmol/L ^ab^ | 1.1 (0.8, 1.4) | 1.0 (0.8, 1.2) | 1.2 (1.0, 1.7) | <0.001 |  | 1.0 (0.8, 1.3) | 2.6 (2.1, 3.4) | <0.001 |
| TC, mmol/L ^ab^ | 4.5 (4.0, 5.0) | 4.3 (3.9, 4.9) | 4.6 (4.1, 5.1) | <0.001 |  | 4.5 (4.0, 5.0) | 4.7 (4.2, 5.4） | 0.001 |
| Hypertension, n(%) | 260 (16.4) | 76 (8.7) | 184 (25.8) | <0.001 |  | 230 (15.6) | 30 (28.3) | 0.001 |
| Diabetes, n(%) | 50 (3.2) | 10 (1.2) | 40 (5.6) | <0.001 |  | 42 (2.8) | 8 (7.6) | 0.007 |
| WC, cm ^ab^ | 80.3 (74.7, 86.0) | 75.3 (71.7, 78.7) | 86.7 (84.0, 90.7) | <0.001 |  | 80.0 (74.3, 85.3) | 86.2 (80.3, 91.3) | <0.001 |
| VAI ^ab^ | 1.4 (1.0, 2.1) | 1.2 (0.9, 1.7) | 1.7 (1.2, 2.4) | <0.001 |  | 1.3 (1.0, 1.9) | 4.2 (3.7, 5.2) | <0.001 |
| LAP ^ab^ | 21.5 (13.4, 33.5) | 14.7 (10.4, 21.5) | 31.8 (23.6, 45.6) | <0.001 |  | 20.1 (13.0, 30.3) | 66.6 (47.2, 92.7) | <0.001 |
| CVAI ^ab^ | 64.3 (34.6, 93.5) | 38.2 (19.8, 57.9) | 95.1 (75.9, 113.7) | <0.001 |  | 61.2 (32.8, 90.3) | 101.2 (77.2, 124.1) | <0.001 |

^a^ Data are median (IQR). ^b^ Average values based on measurements in 2013-2014, 2015, and 2016. NAFLD, non-alcoholic fatty liver disease; BMI, body mass index; SBP, systolic blood pressure; DBP, diastolic blood pressure; FBG, fasting blood glucose; HDL, high-density lipoprotein; LDL, low-density lipoprotein; TG, total cholesterol; TC, total cholesterol; WC, waist circumference; VAI, visceral obesity index; LAP, lipid accumulation; CVAI, Chinese visceral obesity index.
