# Supplementary material for: Associations Between Abdominal Obesity Indices and Nonalcoholic Fatty Liver Disease: Chinese Visceral Adiposity Index
Source: Front Endocrinol (Lausanne). 2022 Mar 10;13:831960. doi: 10.3389/fendo.2022.831960 (PMC8960385; doi:10.3389/fendo.2022.831960)
Supplement: Supplementary file 4 [file Table_2.docx]

Table S2. Basic characteristics according to the LAP and CVAI trajectory patterns from 2013 to 2016.

|  | LAP trajectory groups | | |  | CVAI trajectory groups | | | |
| --- | --- | --- | --- | --- | --- | --- | --- | --- |
|  | Low-rising | High-rising | *P* value |  | Low-rising | Moderate-rising | High-rising | *P* value |
| Variables n (%) | 1501 (94.8) | 83 (5.2) |  |  | 531 (33.5) | 678 (42.8) | 375 (23.7) |  |
| NAFLD n (%) | 339 (22.6) | 68 (81.9) | <0.001 |  | 27 (5.1) | 190 (28.0) | 190 (50.7) | <0.001 |
| Age,year ^ab^ | 38.8 (30.7, 51.9) | 45.8 (32.9, 61.3) | 0.017 |  | 31.7 (28.6, 36.2) | 43.1 (31.9, 51.6) | 58.7 (46.3, 62.7) | <0.001 |
| Male, n (%) | 516 (34.4) | 43 (51.8) | 0.001 |  | 462 (87.0) | 403 (59.4) | 160 (42.7) | <0.001 |
| Current smoking, n (%) | 192 (12.8) | 24 (28.9) | <0.001 |  | 27 (5.1) | 97 (14.3) | 92 (24.5) | <0.001 |
| Current drinking, n (%) | 244 (16.3) | 21 (25.3) | 0.032 |  | 36 (6.8) | 143 (21.1) | 86 (22.9) | <0.001 |
| Income ￥/month, n (%) |  |  | 0.022 |  |  |  |  | <0.001 |
| ≤3000 | 582 (39.5) | 41 (50.0) |  |  | 138 (26.7) | 278 (41.6) | 207 (56.0) |  |
| 3001-5000 | 797 (54.1) | 41 (50.0) |  |  | 348 (67.3) | 343 (51.3) | 147 (39.7) |  |
| ＞5000 | 95 (6.5) | 0 (0.0) |  |  | 31 (6.0) | 48 (7.2) | 16 (4.3) |  |
| Education level, n (%) |  |  | 0.557 |  |  |  |  | <0.001 |
| Illiteracy/primary | 61 (4.1) | 3 (3.6) |  |  | 2 (0.4) | 23 (3.4) | 39 (10.4) |  |
| Middle school | 510 (34.0) | 33 (39.8) |  |  | 85 (16.0) | 258 (38.1) | 200 (53.3) |  |
| College or above | 930 (62.0) | 47 (56.6) |  |  | 444 (83.6) | 397 (58.6) | 136 (36.3) |  |
| BMI, kg/m^2 ab^ | 22.4 (20.8, 24.2) | 26.1 (24.9, 27.7) | <0.001 |  | 20.5 (19.3, 21.8) | 22.9 (21.8, 24.2) | 25.2 (23.9, 26.8) | <0.001 |
| SBP, mmHg ^ab^ | 119.0 (111.3, 128.7) | 134.0 (124.3, 146.0) | <0.001 |  | 111.7 (106.0, 118.7) | 120.8 (114.7, 129.3) | 131.7 (122.7, 143.7) | <0.001 |
| DBP, mmHg ^ab^ | 75.0 (69.7, 82.3) | 82.3 (76.7, 89.3) | <0.001 |  | 71.0 (66.0, 75.3) | 77.0 (71.3, 83.0) | 82.3 (75.0, 89.7) | <0.001 |
| FBG, mmol/L ^ab^ | 5.2 (4.9, 54) | 5.5 (4.9, 5.8) | <0.001 |  | 5.0 (4.8, 5.2) | 5.2 (5.0, 5.4) | 5.4 (5.2, 5.8) | <0.001 |
| HDL, mmol/L ^ab^ | 1.3 (1.1, 1.5) | 1.0 (1.0, 1.2) | <0.001 |  | 1.4 (1.2, 1.5) | 1.2 (1.1, 1.4) | 1.2 (1.0, 1.3) | <0.001 |
| LDL, mmol/L  ^ab^ | 2.5 (2.2, 2.9) | 2.9 (2.6, 3.3) | <0.001 |  | 2.2 (1.9, 2.5) | 2.6 (2.3, 3.0) | 2.9 (2.5, 3.3) | <0.001 |
| TG, mmol/L ^ab^ | 1.1 (0.8, 1.4) | 2.8 (2.3, 3.8) | <0.001 |  | 0.8 (0.7, 1.0) | 1.1 (0.9, 1.5) | 1.5 (1.1, 2.0) | <0.001 |
| TC, mmol/L ^ab^ | 4.4 (3.9, 5.0) | 5.0 (4.6, 5.6) | <0.001 |  | 4.1 (3.7, 4.6) | 4.3 (3.8, 4.8) | 4.8 (4.4, 5.4) | <0.001 |
| Hypertension, n(%) | 225 (15.0) | 35 (42.2) | <0.001 |  | 16 (3.0) | 97 (14.3) | 147 (39.2) | <0.001 |
| Diabetes, n(%) | 39 (2.6) | 11 (13.3) | <0.001 |  | 4 (0.8) | 12 (1.8) | 34 (9.1) | <0.001 |
| WC, cm ^ab^ | 79.7 (74.3, 85.0) | 92.0 (87.0, 95.7) | <0.001 |  | 73.0 (70.0, 76.0) | 81.3 (78.3, 84.7) | 90.0 (86.3, 93.3) | <0.001 |
| VAI ^ab^ | 1.4 (1.0, 1.9) | 3.1 (2.1 5.2) | <0.001 |  | 1.1 (0.9 1.4) | 1.5 (1.1 2.1) | 2.1 (1.5 2.9) | <0.001 |
| LAP ^ab^ | 20.4 (13.1, 30.9) | 79.1 (68.2, 96.5) | <0.001 |  | 11.9 (8.3, 15.6) | 23.7 (17.4, 31.3) | 41.2 (30.8, 56.1) | <0.001 |
| CVAI ^ab^ | 61.3 (33.4, 88.6) | 119.3 (104.0, 139.4) | <0.001 |  | 23.9 (11.1, 34.7) | 70.1 (57.8, 81.8) | 112.7 (102.9, 128.3) | <0.001 |

^a^ Data are median (IQR). ^b^ Average values based on measurements in 2013-2014, 2015, and 2016. NAFLD, non-alcoholic fatty liver disease; BMI, body mass index; SBP, systolic blood pressure; DBP, diastolic blood pressure; FBG, fasting blood glucose; HDL, high-density lipoprotein; LDL, low-density lipoprotein; TG, total cholesterol; TC, total cholesterol; WC, waist circumference; VAI, visceral obesity index; LAP, lipid accumulation; CVAI, Chinese visceral obesity index.
