# Supplementary material for: Associations Between Abdominal Obesity Indices and Nonalcoholic Fatty Liver Disease: Chinese Visceral Adiposity Index
Source: Front Endocrinol (Lausanne). 2022 Mar 10;13:831960. doi: 10.3389/fendo.2022.831960 (PMC8960385; doi:10.3389/fendo.2022.831960)
Supplement: Supplementary file 5 [file Table_3.docx]

Table S3. OR/HR of BMI with Risk of NAFLD.

|  | BMI | |
| --- | --- | --- |
|  | Cross-sectional study | Longitudinal study |
| Model 1 |  |  |
| OR/HR (95%CI) | 1.618 (1.581-1.657) | 3.822 (3.078-4.748) |
| *P* | <0.001 | <0.001 |
| Model 2 |  |  |
| OR/HR (95%CI) | 1.438 (1.403-1.475) | 1.355 (1.003-1.831) |
| *P* | <0.001 | <0.001 |

Model 1, unadjusted model; Model 2, adjusted for age, sex, current smoking, current drinking, income, education level, hypertension,diabetes, and the average measurement of SBP, DBP, FBG, HDL, TC, TG. OR and 95%CI from the cross-sectional study. HR and 95%CI from the longitudinal study.
